# Chromosomal microarray analysis in the genetic evaluation of 279 patients with syndromic obesity

**DOI:** 10.1186/s13039-018-0363-7

**Published:** 2018-02-05

**Authors:** Carla Sustek D’Angelo, Monica Castro Varela, Claudia Irene Emílio de Castro, Paulo Alberto Otto, Ana Beatriz Alvarez Perez, Charles Marques Lourenço, Chong Ae Kim, Debora Romeo Bertola, Fernando Kok, Luis Garcia-Alonso, Celia Priszkulnik Koiffmann

**Affiliations:** 1 Human Genome and Stem Cell Research Center (HUG-CELL), Department of Genetics and Evolutionary Biology, Institute of Biosciences, University of Sao Paulo, Rua do Matao no 277, Cidade Universitaria-Butanta, Sao Paulo, SP 05508-090 Brazil; 2 Department of Morphology and Genetics, Paulista School of Medicine, Federal University of Sao Paulo (UNIFESP), Sao Paulo, SP Brazil; 3 Neurogenetics Unit, Clinics Hospital of Ribeirao Preto, Faculty of Medicine, University of Sao Paulo, FMRP-USP, Ribeirao Preto, SP Brazil; 4 Genetic Unit, Children’s Institute, Faculty of Medicine, University of Sao Paulo, FMUSP, Sao Paulo, SP Brazil; 5 Department of Neurology, Faculty of Medicine, University of Sao Paulo, FMUSP, Sao Paulo, SP Brazil

**Keywords:** Chromosomal microarray analysis (CMA), Copy number variations (CNVs), Body mass index (BMI), Intellectual and developmental disabilities (IDDs), Prader-Willi syndrome (PWS), Syndromic obesity

## Abstract

**Background:**

Syndromic obesity is an umbrella term used to describe cases where obesity occurs with additional phenotypes. It often arises as part of a distinct genetic syndrome with Prader-Willi syndrome being a classical example. These rare forms of obesity provide a unique source for identifying obesity-related genetic changes. Chromosomal microarray analysis (CMA) has allowed the characterization of new genetic forms of syndromic obesity, which are due to copy number variants (CNVs); however, CMA in large cohorts requires more study. The aim of this study was to characterize the CNVs detected by CMA in 279 patients with a syndromic obesity phenotype.

**Results:**

Pathogenic CNVs were detected in 61 patients (22%) and, among them, 35 had overlapping/recurrent CNVs. Genomic imbalance disorders known to cause syndromic obesity were found in 8.2% of cases, most commonly deletions of 1p36, 2q37 and 17p11.2 (5.4%), and we also detected deletions at 1p21.3, 2p25.3, 6q16, 9q34, 16p11.2 distal and proximal, as well as an unbalanced translocation resulting in duplication of the *GNB3* gene responsible for a syndromic for of childhood obesity. Deletions of 9p terminal and 22q11.2 proximal/distal were found in 1% and 3% of cases, respectively. They thus emerge as being new putative obesity-susceptibility *loci*. We found additional CNVs in our study that overlapped with CNVs previously reported in cases of syndromic obesity, including a new case of 13q34 deletion (*CHAMP1*), bringing to 7 the number of patients in whom such defects have been described in association with obesity. Our findings implicate many genes previously associated with obesity (e.g. *PTBP2*, *TMEM18*, *MYT1L*, *POU3F2*, *SIM1*, *SH2B1*), and also identified other potentially relevant candidates including *TAS1R3*, *ALOX5AP*, and *GAS6.*

**Conclusion:**

Understanding the genetics of obesity has proven difficult, and considerable insight has been obtained from the study of genomic disorders with obesity associated as part of the phenotype. In our study, CNVs known to be causal for syndromic obesity were detected in 8.2% of patients, but we provide evidence for a genetic basis of obesity in as many as 14% of cases. Overall, our results underscore the genetic heterogeneity in syndromic forms of obesity, which imposes a substantial challenge for diagnosis.

**Electronic supplementary material:**

The online version of this article (10.1186/s13039-018-0363-7) contains supplementary material, which is available to authorized users.

## Background

Obesity is a highly heritable multifactorial disorder defined by a body mass index (BMI) of ≥30 kg/m^2^, which predisposes to many diseases. Rare and common genetic variants associated with obesity identified to date have increased our understanding of the mechanisms by which obesity develops. Copy number variants (CNVs) in a number of chromosomal regions are known to be involved in highly penetrant and individually rare, both isolated and syndromic forms of obesity [1–3]. The latter describes cases where obesity co-occurs with additional phenotypes (e.g., intellectual and developmental disabilities (IDDs), dysmorphism, congenital anomalies) often arising as part of a distinct syndrome, from which Prader-Willi syndrome (PWS; OMIM #176270) is a classical example. Until recently, only a few genomic disorders other than PWS were known to contribute to increased risk of obesity, including the known microdeletion syndromes 1p36 (OMIM #607872), 2q37 (OMIM #600430), 6q16 (*SIM1* gene), 9q34 (OMIM #610253; *EHMT1* gene), 11p14.1 (OMIM #612469), and 17p11.2 (OMIM #182290; *RAI1* gene).

In recent years, however, numerous unique and rare recurring/overlapping CNVs have been associated with a syndromic obesity phenotype in patients through the widespread use of chromosomal microarray analysis (CMA) [4–16]. Examples include deletions of chromosome band 2p25.3 that include the *MYT1L* gene (OMIM #616521), the recurrent 220-kb deletion of distal 16p11.2 including the *SH2B1* gene (OMIM #613444), and the recurrent 600-kb 16p11.2 proximal deletion (OMIM #611913, gene unknown). Also, a novel genomic disorder that causes obesity, ID and seizures has been described in children carrying a recurrent unbalance translocation (8;12)(p23.1;p13.31) that duplicates the *GNB3* gene [17]. Overlapping 1p21.3 deletions comprising the *DPYD* and *MIR137* genes have been detected in patients with a phenotype consisting primarily of obesity, ID, and autism spectrum disorder (ASD) [18, 19]. Small 6q16.1 deletions encompassing the *POU3F2* gene were identified in 10 individuals presenting with obesity, hyperphagia and IDDs [20]. Chromosome 13q34 deletions disrupting the *CHAMP1* gene were linked ID, obesity and mild dysmorphism in five adult individuals [21].

Syndromic obesity is recognized as an etiologically heterogeneous group of disorders for which an obesity-related genetic change can be identified but only a few genetic causes have been identified to date. Only one study has examined the etiology of syndromic obesity with CMA in a cohort of 100 patients specifically selected for obesity [22]. In that study, CNVs were regarded either as pathogenic or potentially pathogenic in 22% of cases, and several novel CNVs for which a defined syndrome has not yet been delineated were uncovered. Herein, we report our experience over the past 5 years using CMA to identify CNVs in 279 patients referred with syndromic obesity. This study adds to the current knowledge of CNVs linked to obesity and provides evidence for association with obesity at new and previously identified candidate *loci*.

## Methods

### Cohort enrollment and description

Only patients who tested negative for PWS (methylation analysis of *SNURF-SNRPN* exon 1 by our laboratory) were included in our study whether or not they had a positive clinical score for PWS. This test population had a mean age of 9 years (range 8 days to 40 years old), 55% of cases represented by male patients (male/female ratio = 1.2). The 2000 Centers for Disease Control and Prevention (CDC) growth charts (available at https://www.cdc.gov/growthcharts/) were used to plot weight-for-age, height-for-age, weight-for-height, BMI-for-age, and occipito-frontal head circumference (OFC) [23]. We stratified our cohort into 4 age groups: (1) infants (*n* = 19) < 2 years old (mean age 13 months; 9 males and 9 females); (2) children (*n* = 153) aged 2–9 years (mean age 6 years; 80 males and 73 females); (3) adolescents (*n* = 98) aged 10–19 years (mean age 9 years; 61 males and 37 females); (4) adults (*n* = 10) > 20 years old (mean age 27 years; 2 males and 8 females In a majority of patients, recognition of excessive weight gain was based on the following: (1) infants, the standard deviation (SD) of weight-for-height Z-scores > ~ 1 (mean + 3.23 SD); (2) children and adolescents, BMI-for-age percentiles ≥85th (mean 97.4th and 97.9th percentiles, respectively); (3) adults, BMI values ≥30 kg/m2 (mean 46.7 kg/m2); In 37 patients, data on weight and/or height were missing but they had a documented diagnosis of overweight or obesity made by attending physicians, and 5 other patients aged < 5 years had hyperphagia with an increase probability of developing obesity. Among a subset of 208 children and adolescents (BMI ≥ 95th percentile), we further classified obesity based on the BMI expressed as a percentage above the 95th BMI percentile according to age and sex, as previously described [24, 25]: a BMI 100–119% of the 95th percentile was used to define moderate obesity and a BMI ≥120% of the 95th BMI percentile used to define severe obesity. Extreme BMIs were calculated by multiplying the BMI at the 95th percentile by a factor of 1.1 through 1.9 to derive the 110% to 190%, for both genders.

### Chromosomal microarray analysis

Any of the following genome-wide array platforms were used according to their availability: CytoSure ISCA v2 4x180K (Oxford Gene Technology, Oxford, UK), SurePrint G3 Human CGH 8x60K (Agilent Technologies, Santa Clara, CA), Affymetrix Mapping 100 K and 500 K arrays (Affymetrix, Santa Clara, CA, USA). Most cases (85%) were investigated using high-density oligonucleotide microarrays (4x180K OGT platform). DNA was extracted from peripheral blood using Autopure LS® (Gentra Systems, Inc., Minneapolis, MN). Genomic DNA concentration was measured by Nanodrop spectrophotometer (ThermoFisher). Chromosomal microarray testing was performed according to the manufacturers’ instructions. In oligonucleotide-based microarrays, two experiments were performed for each patient sample with reversal of the dye labels for the control and test samples, raw data were processed and analyzed using Agilent Feature Extraction and Genomic Workbench software with the statistical algorithm ADM-2 and sensitivity threshold of 6.7. Affymetrix SNP array data was analyzed with the Genotyping Console (GTC) 4.0 software using default settings and a similarly processed reference sample data set. Due to the limited probe coverage, CNVs on chromosome Y were removed from the analysis. We used the American College of Medical Genetics and Genomics (ACMG) 2011 guidelines for variant interpretation to classify variants in 4 categories: pathogenic CNVs (PCNVs), likely PCNVs, variants of uncertain significance (VUS), and likely benign CNVs [26]. Healthy and disease variant databases used included the Database of Genomic Variants (DGV, http://dgv.tcag.ca), the Online Mendelian Inheritance in Man (OMIM, https://www.omim.org/) and the DatabasE of genomiC varIation and Phenotype in Humans using Ensembl Resources (DECIPHER, http://decipher.sanger.ac.uk) [27–29]. All genomic breakpoints were based on the human genome build GRCh37 (hg19) (http://genome.ucsc.edu/) [30].

### Gene prioritization

Genes affected by the detected CNVs were compared to a list of genes related to obesity downloaded from the Text-mined Hypertension, Obesity and Diabetes candidate gene database (T-HOD) [31] and the Human Genome Epidemiology encyclopedia Navigator (HUGE, https://phgkb.cdc.gov/PHGKB) [32]. We specifically searched the term “obesity” and retrieved 835 genes annotated in T-HOD and 1920 genes annotated in the HUGE Phenopedia. We also checked the genes affected by CNVs against a list of 370 genes with evidence for playing a role in obesity curated from literature [33] and a list of 940 genes in the CNV morbidity map for IDDs generated from 29,085 cases and 19,584 controls [34].

## Results

### Cohort and correlation of PCNVs with specific phenotypes

**Fig. 1:**
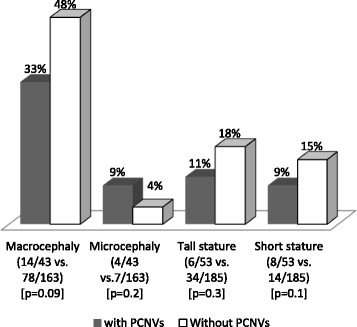
Comparison of frequencies of each variable among patients with and without PCNVs. The fractions in parentheses indicate the number of cases that manifested the phenotype over the total number of cases with data. *P*-values for the Fisher’s exact test are shown. Macro/microcephaly and tall/short stature were defined by the Z-scores > or < ±2 SD

General clinical findings noted in patients are listed in Table 1 (individual descriptions are provided in Table S1 in Additional file 1). Although patients’ records were not always complete and clinical comorbidities could not be fully assessed, the most commonly reported features associated with obesity were IDDs, dysmorphism, behavioral phenotypes, hyperphagia, neonatal hypotonia, and language impairments. Hands and feet abnormalities, abnormalities of the external genitalia and eye/vision problems were often reported. Macrocephaly was observed in 92 of 206 (45%) patients, compared to 11 of 206 (5%) patients with microcephaly, and tall stature in 40 of 238 (17%) patients, compared to 22 of 238 (9%) patients with short stature (Z-scores > or < ±2 SD). No association was found between these growth parameters in cases with and without PCNVs using the Fisher’s Exact test (Fig. 1).

**Table 1:** Additional phenotypes of patients with syndromic obesity

Clinical features	Total cohort (n)	Patients with PCNVs (n)
Intellectual/developmental disabilities	219	55
Craniofacial dysmorphism	149	49
Behavioral problems	132	29
Hyperphagia	112	27
Infantile hypotonia	88	32
Language impairments	80	28
Hands/ft abnormalities	65	21
Abnormal external genitalia	56	20
Eye/vision problems	51	16
Seizures	31	13
Poor motor skills	29	9
Skeletal anomalies	23	9
Brain abnormalities	19	7
Hearing loss	13	6
Cardiac abnormalities	10	4

Prevalence could not be assess as complete phenotypic data was not available

**Fig. 2:**
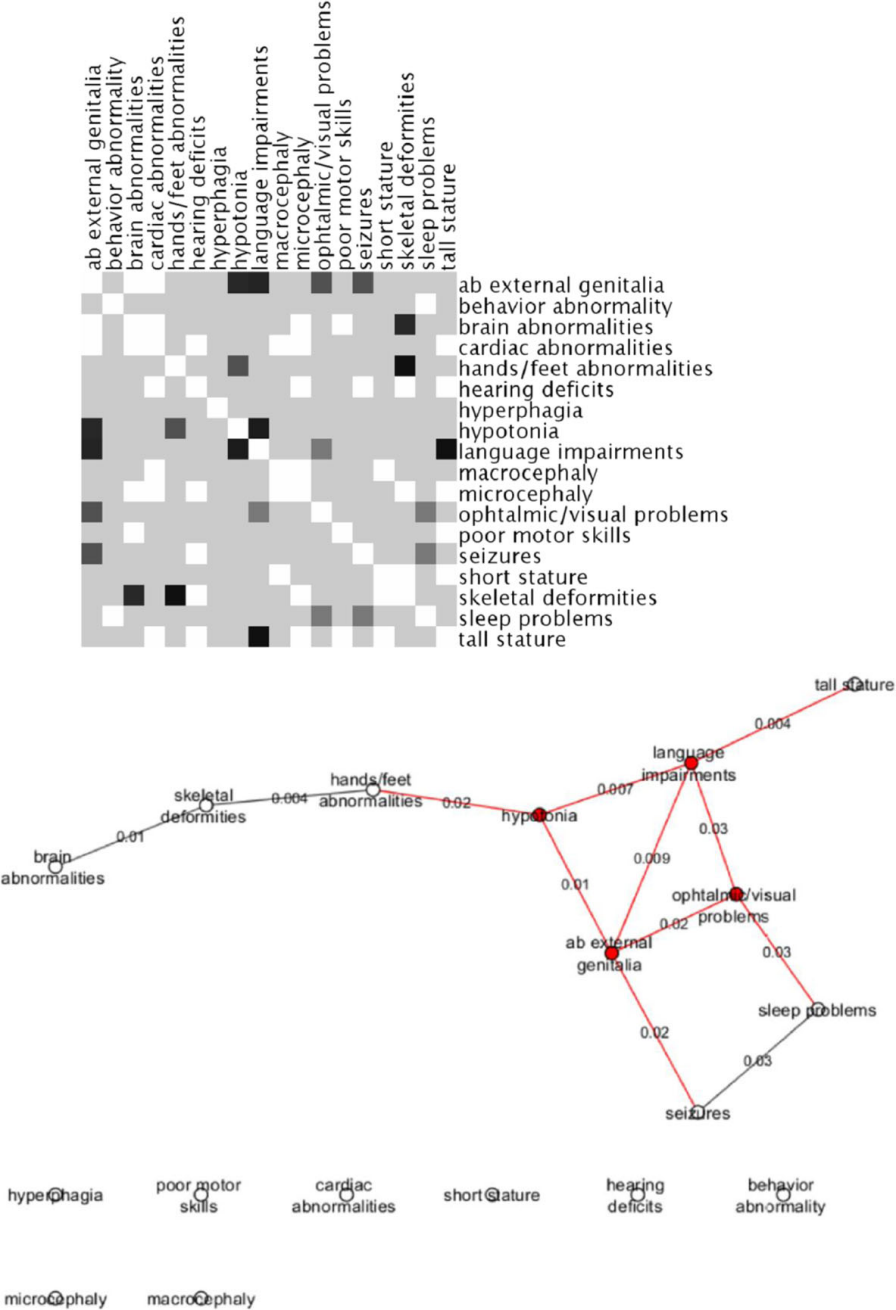
The heat map constructed from the *P-*values for the Chi-square statistic test between pairs of phenotypes observed in patients with PCNVs against those without PCNVs is shown at the top, where *P*-values < 0.05 are represented by small darker gray or black squares and larger values by light gray squares (white squares indicate null values or the absence of association between a given pair of phenotype). Graphical representation of the phenotype network generated using Cytoscape is shown at the bottom, where phenotypes (nodes) are interconnected (edges) if they had significant associations for PCNVs at *P* < 0.05. The resulting network has 10 interconnected phenotypes and 8 not connected phenotype variables. Red nodes and edges highlight the most highly connected phenotypes and their interactions in the network

In an attempt to determine whether there were phenotypic differences associated with the presence of PCNVs, we compared the frequencies of phenotype pairs segregating together in patients with syndromic obesity caused by PCNVs against those without PCNVs. We constructed a matrix representation (heatmap) of the Chi-square *p*-values between any given pair of phenotypes that co-occurred in patients with and without PCNVs. Out of 133 phenotype pairs that were evaluated (listed in Table S2 in Additional file 2), 12 had significant associations for PCNVs with *p-*values < 0.05 (Fig. 2a). Next, we constructed using Cytoscape [35] a graphic representation of these phenotype-phenotype associations, where a given pair of phenotype was connected only if they were significant at *p* < 0.05, to discover the core phenotype variables in the network (those that overlapped most between pairs). The most highly correlated phenotypes are hypotonia, language impairments, abnormalities of the external genitalia, and eye/vision problems. They also correlated with many additional phenotype variables including seizures, sleep problems, tall stature and hands/feet abnormalities (Fig. 2b).

### Characterization of CNVs in patients with syndromic obesity

Overall, CMA identified clinically relevant genomic imbalances in 22% of patients, potentially clinically relevant CNVs in 2%, VUS in 5% and likely benign CNVs in 11% (Table 2). All clinically relevant results per individual are listed in Table 3. The genomic regions associated with likely PCNVs, VUS and likely benign CNVs are listed in Additional file 3: Table S3.

**Table 2:** Overall findings of microarray testing

	Number	%
Total number of cases	279	–
With imbalances	112	40
With pathogenic CNVs	61	22
*With Known syndromes*	*47*	*17*
Pathogenic imbalances ^a^	68	–
Del	49	72
Dup	19	28
> 5 Mb	21	31
De novo	30	49
Inherited	6	10
Unknown	19	31
Not maternal	6	10
With likely pathogenic CNVs	6	2
With CNVs of uncertain significance	15	5
With likely benign CNVs	30	11

^a^Pathogenic imbalances included 45 simple deletions or duplications, 4 unbalanced translocations, an insertional translocation, and 6 other complex rearrangements believed to have been formed from the same rearrangement. In the remaining 5 patients, the rearrangements were associated with a second-site CNV arisen apparently independent which were classified as benign or of uncertain significance

**Table 3:** Pathogenic copy number variations (PCNVs) detected in 279 patients with syndromic obesity

Case number	Age	Gender	Weight Status	CNV Type	Cytoband	Genome Coordinate	Size	Origin	Clinical significance	RefSeq genes
*Genomic imbalance disorders*										
**P1**	5y	F	Referred as obese	Del	1p36.33	734595-1970865	1236270	Unk	1p36 terminal deletion	***KLHL17*** *,* AGRN *,* TAS1R3 *,* * DVL1 *,* ***VWA1*** *,* ***MMP23B*** *,* ***GABRD***
**P2**	34y	F	BMI 59.1	Del	1p36.33	734595-2223317	1488722	Unk	1p36 terminal deletion, complex	***KLHL17*** *,* AGRN *,* TAS1R3 *,* * DVL1 *,* ***VWA1*** *,* ***MMP23B, GABRD,*** PRKCZ *,* SKI *
				Dup	1p36.33 p36.32	2225679-2694799	469120			--
**P3**	14y	F	BMI 26.8 (93.3th)	Del	1p36.33 p36.32	794592-2377269	1582677	De novo	1p36 terminal deletion	***KLHL17*** *,* AGRN *,* TAS1R3 *,* * DVL1 *,* ***VWA1*** *,* ***MMP23B*** *,* ***GABRD,*** PRKCZ *,* SKI *
**P4**	6y	F	BMI 19.7 (96.8th)	Del	1p36.33 p36.32	734595-3531040	2796445	Unk	1p36 terminal deletion	***KLHL17*** *,* AGRN *,* TAS1R3 *,* * DVL1 *,* ***VWA1*** *,* ***MMP23B*** *,* ***GABRD,*** PRKCZ *,* SKI ,* TNFRSF14 *,* PRDM16 *
**P5**	11y	M	BMI 30.8 (99th)	Del	1p36.31 p36.22	6204969-9433118	3228149	Unk	1p36 interstitial deletion	CAMTA1 *,* PER3 *,* UTS2 *, RERE,* H6PD *
**P6**	15y	F	BMI 37.1 (99.1th)	Del	1p22.1 p21.2	93919217-99846176	5926959	De novo	1p21.3 deletion	F3 *,* PTBP2 *,* * DPYD *,* ***MIR137***
**P7**	8y	F	BMI 33.2 (99.7th)	Del	1p21.3 p13.3	95696444-107755879	12059435	Unk	1p21.3 deletion	PTBP2 *,* * DPYD *,* ***MIR137*** *,* VCAM1 *,* * COL11A1 *,* AMY2B *,* AMY2A *,* AMY1A *
**P8**	6y	M	BMI 28.8 (99.9th)	Dup	1q21.1	146074084-147828029	1753945	Unk	1q21.1 distal duplication	PRKAB2 *,* ***CHD1L*** *,* ***GJA5*** *,* ***GJA8***
**P9**	7y	F	BMI 22.6 (98.2th)	Del	2p25.3	63452-3215593	3152141	De novo	2p25.3 terminal deletion	ACP1 *,* * TMEM18 *,* * SNTG2 *,* TPO *,* * MYT1L
**P10**	8y	F	BMI 29.1 (99.5th)	Del	2q37.2 q37.3	237220842-242995835	5774993	De novo	2q37 terminal deletion	PRLH *,* LRRFIP1 , PER2 *,* ***HDAC4*** *,* GPC1 *,* * CAPN10 *,* * GPR35 *,* * KIF1A *,* PASK *,* STK25 *
**P11**	21y	M	Referred as obese	Del	2q37.2 q37.3	236854160-242995835	6141675	Unk	2q37 terminal deletion	***AGAP1*** *,* PRLH *,* LRRFIP1 , PER2 *,* ***HDAC4*** *,* GPC1 *,* * CAPN10 *,* * GPR35 *,* * KIF1A *,* PASK *,* STK25 *
**P12**	10y	M	BMI 29.8 (99th)	Del	2q37.2 q37.3	236944801-243014630	6069829	De novo	2q37 terminal deletion, complex	***AGAP1*** *,* PRLH *,* LRRFIP1 , PER2 *,* ***HDAC4*** *,* GPC1 *,* * CAPN10 *,* * GPR35 *,* * KIF1A *,* PASK *,* STK25 *
				Dup	2q37.1 q37.2	235090417-236802930	1712513			--
**P13**	9y	F	BMI 24.7 (98.2th)	Del	2q37.1 q37.3	234850276-243028335	8178059	Unk	2q37 terminal deletion, complex	TRPM8 ***, AGAP1*** *,* PRLH *,* LRRFIP1 , PER2 *,* ***HDAC4*** *,* GPC1 *,* * CAPN10 *,* * GPR35 *,* * KIF1A *,* PASK *,* STK25 *
				Dup	2q37.1	233867403-234794816	927413			UTG1A1 *
**P14**	5y	F	BMI 24.0 (99.7th)	Del	2q37.3	240880562-242948060	2067498	Unk	Unbalanced translocation	GPC1 *,* * CAPN10 *,* * GPR35 *,* * KIF1A *,* PASK *,* STK25 *
				Dup	17q25.3	78709250-81036261	2327011			RPTOR *,* ACTG1 *,* GCGR *,* * PCYT2 *,* FASN *,* * CSNK1D *,* UTS2R
**P15**	10y	M	BMI 31.0 (99.3th)	Del	6q16.1 q21	95836632-108010940	12174308	De novo	6q16 deletion	KLHL32 *, POU3F2,* MCHR2 *,* * SIM1 *,* * GRIK2 *,* LIN28B *,* * ATG5
**P16**	15y	F	BMI 40.5 (99.4th)	Del	7q11.23	72420782-74985644	2564862	Unk	7q11.23 deletion	***FKBP6*** *,* ***FZD9*** *,* BCL7B *,* TBL2 *,* MLXIPL *,* STX1A *,* * CLDN3 *,* ***ELN*** *,* ***LIMK1*** *,* RFC2 *,* * CLIP2 *,* ***GTF2IRD1*** *,* ***GTF2I*** *,* ***NCF1***
**P17**	6y	M	BMI 30.6 (99.9th)	Del	7q11.23	72437606-75053787	2616181	Unk	7q11.23 deletion	***FKBP6*** *,* ***FZD9*** *,* BCL7B *,* TBL2 *,* MLXIPL *,* STX1A *,* * CLDN3 *,* ***ELN*** *,* ***LIMK1*** *,* RFC2 *,* * CLIP2 *,* ***GTF2IRD1*** *,* ***GTF2I*** *,* ***NCF1***
**P18**	6y	M	BMI 33.4 (99.9th)	Del	8p23.3 p23.1	176464-7786759	7610295	De novo	Unbalanced translocation	***CLN8*** *,* CSMD1 *,* DEFA1 *,* * DEFB103A *,* ***DEFB103B*** *,* ***DEFB104A*** *,* ***DEFB106A*** *,* ***DEFB105A*** *,* ***DEFB107A*** *,* ***DEFB4A***
				Dup	12p13.33 p13.31	204618-8309473	8104855			***SLC6A13*** *,* WNT5B *,* * ADIPOR2, * CACNA2D4 *,* ***CACNA1C*** *,* FOXM1 *,* TEAD4 *,* PARP11 *,* ***KCNA1*** *,* NTF3 *,* VWF *,* TNFRSF1A *,* * SCNN1A *,* GAPDH *,* CD4 *,* GNB3 *,* * CD163 *,* APOBEC1 *,* GDF3 *,* SLC2A14 *,* C3AR1
**P19**	6y	M	BMI 25.8 (99.8th)	Dup	8p23.1	8054556-11985356	3930800	Not mat	8p23.1 duplication	***CLDN23*** *,* MFHAS1 *,* PPP1R3B *,* LOC157273 *,* TNKS *,* * MSRA *,* * SOX7 *,* MTMR9 *,* * BLK *,* GATA4 *,* * NEIL2 *,* FDFT1 *,* * CTSB
				Dup	13q12.12	23706634-24910765	1204131	Not mat	Uncertain	*SGCG,* ^***¶***^ ***SACS,*** *MIPEP*
**P20**	11y	F	BMI 26.7 (97.6th)	Del	9p24.3 p22.3	40910-14304973	14264063	De novo	9p terminal deletion	***KANK1*** *,* ***DMRT1*** *,* SMARCA2 *,* VLDLR *,* * GLIS3 *,* JAK2 *,* ***RLN1*** *,* IL33 *,* * KDM4C *,* PTPRD *
**P21**	12m	F	Weight-for Height +1sd	Del	9p24.3 p22.3	204149-15260439	15056290	De novo	9p terminal deletion	***KANK1*** *,* ***DMRT1*** *,* SMARCA2 *,* VLDLR *,* * GLIS3 *,* JAK2 *,* ***RLN1*** *,* IL33 *,* * KDM4C *,* PTPRD, * TTC39B
**P22**	17y	M	BMI 28.7 (95.7th)	Del	9p24.3 p22.3	201149-8807593	8606444	Unk	9p terminal deletion	***KANK1*** *,* ***DMRT1*** *,* SMARCA2 *,* VLDLR *,* * GLIS3 *,* JAK2 *,* ***RLN1*** *,* IL33 *,* * KDM4C *,* PTPRD *
**P23**	9y	F	BMI 31.1 (99.5th)	Del	9q34.3	140665414-141018984	353570	De novo	9q34.3 deletion	EHMT1 *,* * CACNA1B
**P24**	14y	M	BMI 28.7 (97.6th)	Del	13q12.3 q13.1	29081250-33529310	4448060	De novo	13q12.3 deletion	*POMP* , *SLC46A3* , *MTUS2* , *SLC7A1* , *UBL3,* KATNAL1 *, LINC00426, HMGB1,* ***ALOX5AP*** *,* * *RXFP2,* BRCA2*
**P25**	8y	M	BMI 25.4 (99.2th)	Del	15q11.2	22729423-23086969	357546	Unk	15q11.2 microdeletion	***NIPA1*** *,* ***NIPA2*** *,* ***CYFIP1***
**P26**	10y	F	BMI 22.1 (93.4th)	Del	16p13.12 p13.11	14780302-16400774	1620472	Pat	16p13.1 deletion	NDE1 *,* MYH11 *,* * ABCC1 *,* ABCC6
**P27**	4y	F	Hyperphagia	Dup	16p13.12 p13.11	14796004-16586941	1790937	Pat	16p13.1 duplication	NDE1 *,* MYH11 *,* * ABCC1 *,* ABCC6
**P28**	7y	F	BMI 20.5 (96.6th)	Del	16p11.2	28843754-29044850	201096	De novo	16p11.2 (BP 2-3) deletion	ATXN2L *,* * TUFM *,* * MIR4721 *,* SH2B1 *,* * ATP2A1 ,* SPNS1
**P29**	12y	M	BMI 30.4 (98.8th)	Del	16p11.2	29592751-30197466	604715	Not mat	16p11.2 (BP 4-5) deletion	***QPRT*** *,* ***PRRT2*** *,* ***SEZ6L2*** *,* ***DOC2A*** *,* ***ALDOA*** *,* ***TBX6*** *,* MAPK3
**P30**	8y	M	Referred as obese	Dup	16p11.2	29592751-30197466	604715	Pat	16p11.2 (BP 4-5) duplication	***QPRT*** *,* ***PRRT2*** *,* ***SEZ6L2*** *,* ***DOC2A*** *,* ***ALDOA*** *,* ***TBX6*** *,* MAPK3
**P31**	11y	M	BMI 42.2 (99.7th)	Del	17p11.2	17006987-20171357	3164370	De novo	17p11.2 deletion	***COPS3*** *,* ***NT5M*** *,* ***MED9*** *,* PEMT *,* * RAI1 *,* SREBF1 *,* * ATPAF2 *,* ***DRG2*** *,* ***SMCR8*** *,* MFAP4 *,* SLC47A1 *,* ALDH3A2, ***SPECC1***
**P32**	6y	M	BMI 31.2 (99.9th)	Del	17p11.2	16757563-20395535	3637972	Not mat	17p11.2 deletion	TNFRSF13B *,* ***COPS3*** *,* ***NT5M*** *,* ***MED9*** *,* PEMT *,* * RAI1 *,* SREBF1 *,* * ATPAF2 *,,* ***DRG2*** *,* ***SMCR8*** *,* MFAP4 *,* SLC47A1 *,* ALDH3A2, ***SPECC1***
**P33**	7y	F	BMI 21.9 (97.9th)	Del	17p11.2	16603145-20395535	3792390	De novo	17p11.2 deletion	TNFRSF13B *,* ***COPS3*** *,* ***NT5M*** *,* ***MED9*** *,* PEMT *,* * RAI1 *,* SREBF1 *,* * ATPAF2 *,* ***DRG2*** *,* ***SMCR8*** *,* MFAP4 *,* SLC47A1 *,* ALDH3A2, ***SPECC1***
**P34**	8y	F	BMI 25.9 (98.9th)	Del	17p11.2	16603145-20395535	3792390	Unk	17p11.2 deletion	TNFRSF13B *,* ***COPS3*** *,* ***NT5M*** *,* ***MED9*** *,* PEMT *,* * RAI1 *,* SREBF1 *,* * ATPAF2 *,* ***DRG2*** *,* ***SMCR8*** *,* MFAP4 *,* SLC47A1 *,* ALDH3A2, ***SPECC1***
**P35**	10y	M	BMI 28.1 (99th)	Del	17p11.2	16603145-20463399	3860254	De novo	17p11.2 deletion	TNFRSF13B *,* ***COPS3*** *,* ***NT5M*** *,* ***MED9*** *,* PEMT *,* * RAI1 *,* SREBF1 *,* * ATPAF2 *,* ***DRG2*** *,* ***SMCR8*** *,* MFAP4 *,* SLC47A1 *,* ALDH3A2, ***SPECC1***
**P36**	9y	M	BMI 22.2 (96.8th)	Dup	17q21.31 q21.32	40993738-45166786	4173048	De novo	17q21.3 duplication	AOC3 *,* G6PC *,* * BRCA1 *,* * SOST *,* * PPY *,* * PYY *,* * TMEM101 *,* HDAC5 *,* ITGA2B *,* ***EFTUD2*** *,* PLCD3 *,* CRHR1 *,* * MAPT *,* ***KANSL1***
**P37**	8y	F	BMI 22.3 (97.4th)	Dup	19p13.2	12640509-13231703	591194	Unk	19p13.2 duplication	*MAST1, CALR,* ***NFIX***
				Dup	9p22.1	19066513-19497724	431211	Unk	Uncertain	PLIN2 *
**P38**	18y	F	BMI 41.5 (99th)	Del	19p13.12	14384925-16034584	1649659	De novo	19p13.12 deletion	*CD97, DDX39A* ***,*** *PKN1* , *PTGER1, GIPC1, CASP14,* ***NOTCH3,*** CYP4F11
**P39**	8y	F	Referred as obese	Del	22q11.21	18890162-20311554	1421392	De novo	22q11.2 deletion	***PRODH*** *,* ***DGCR2*** *,* ***DGCR14*** *,* ***CDC45*** *,* TBX1 *,* * GNB1L *,* TXNRD2 *,* COMT *,* * DGCR8 *,* ***ZDHHC8***
**P40**	3y	M	BMI 34.7 (99.9th)	Del	22q11.21	18661758-21684798	3023040	De novo	22q11.2 deletion	***PRODH*** *,* ***DGCR2*** *,* ***DGCR14*** *,* ***CDC45*** *,* TBX1 *,* * GNB1L *,* TXNRD2 *,* COMT *,* * DGCR8 *,* ***ZDHHC8*** *,* ***PI4KA,*** SLC74A
**P41**	9y	M	BMI 32.5 (99.6th)	Del	22q11.21	18661758-21684798	3023040	De novo	22q11.2 deletion	***PRODH*** *,* ***DGCR2*** *,* ***DGCR14*** *,* ***CDC45*** *,* TBX1 *,* * GNB1L *,* TXNRD2 *,* COMT *,* * DGCR8 *,* ***ZDHHC8*** *,* ***PI4KA,*** SLC74A
**P42**	13m	M	Weight-for Height +1sd	Del	22q11.21	18818429-21661436	2843007	Pat	22q11.2 deletion	***PRODH*** *,* ***DGCR2*** *,* ***DGCR14*** *,* ***CDC45*** *,* TBX1 *,* * GNB1L *,* TXNRD2 *,* COMT *,* * DGCR8 *,* ***ZDHHC8*** *,* ***PI4KA,*** SLC74A
**P43**	18y	M	Referred as obese	Dup	22q11.21	18890162-21464056	2573894	Pat	22q11.2 duplication	***PRODH*** *,* ***DGCR2*** *,* ***DGCR14*** *,* ***CDC45*** *,* TBX1 *,* * GNB1L *,* TXNRD2 *,* COMT *,* * DGCR8 *,* ***ZDHHC8*** *,* ***PI4KA,*** SLC74A
**P44**	5y	M	BMI 27.9 (99.9th)	Del	22q11.21 q11.23	21759572-23822925	2063353	Unk	22q11.2 deletion, distal	***HIC2,*** MAPK1, * GNAZ *,* ***BCR***
**P45**	7y	F	BMI 24.8 (98.9th)	Del	22q11.21 q11.22	21468437-22959609	1491172	Not mat	22q11.2 deletion, distal	***HIC2*** *,* MAPK1 *
				Dup	3p26.3	857110-1414719	557609	Not mat	Uncertain	--
**P46**	2y	F	BMI 17.7 (85th)	Del	22q11.22 q11.23	23012069-23648827	636758	Mat	22q11.2 deletion, distal	***GNAZ*** *,* ***BCR***
**P47**	15y	M	BMI 39.5 (99.6th)	Del	22q11.22 q11.23	23063178-23696464	633286	Unk	22q11.2 deletion, distal	***GNAZ*** *,* ***BCR***
*Other pathogenic imbalances*										
**P48**	2y	M	BMI 24.4 (99.9th)	Del	3p26.3	73603-1273300	1199697	De novo	Unbalanced translocation	***CHL1***
				Dup	11q22.3 q25	106251478-134668665	28417187			ACAT1 *,* ATM *,* POU2AF1 *,* IL18 *,* * ANKK1 *,* DRD2 *,* * HTR3B, HTR3A *,* NNMT *,* BUD13 *,* APOA5 *,* * APOA4 *,* * APOC3 *,* * APOA1 *,* * BACE1 *,* IL10RA *,* CD3E *,* HYOU1 *,* H2AFX *,* CBL *,* * USP2 *,* THY1 *,* ARHGEF12 *,* BSX *,* * HSPA8 *,* CLMP *,* ***NRGN*** *,* SLC37A2 *,* TIRAP *,* ***KCNJ1*** *,* KCNJ5 *,* * OPCML
**P49**	14y	F	Referred as obese	Del	3p24.1	28719852-30169971	1450119	Unk	Complex rearrangement	*LINC00693, RBMS3-AS3, RBMS3* , *RBMS3-AS1*
				Dup	3q11.2 q13.31	93558505-115890384	22331879			EPHA6 *,* ARL6 *,* * STG3GAL6 *,* ***COL8A1*** *,* CCDC80 *,* BOC *,* ZDHHC23 *, ZBTB20* , *GAP43, LSAMP,* DRD3
**P50**	10y	M	BMI 30.4 (99.3th)	Del	3q25.33	159252702-160555217	1302515	De novo	Uncertain	IL12A
				Del	13q31.2 q32.1	89522636-95065310	5542674	De novo	Feingold syndrome	***MIR17HG*** *,* GPC5 *,* * GPC6
**P51**	14y	F	BMI 27.9 (95.8th)	Del	7q22.1 q22.3	102358320-105487655	3129335	De novo	Clinically relevant	NAPEPLD *,* ***RELN*** *,* LHFPL3
**P52**	2y	F	BMI 27.0 (99.9th)	Del	10p15.3 p14	269695-11579546	11309851	De novo	Unbalanced translocation	***ZMYND11*** *,* DIP2C *,* * IDI1 *,* ADRAB2 *,* PFKP *,* * KLF6 *,* ARK1C1 *,* AKR1C2 *,* ARK1C3 *,* AKR1C4 *,* UCN3 *,* IL15RA *,* IL2RA *,* * PFKFB3 *,* PRKCQ *,* ***GATA3***
				Dup	6q27	169505179-170694486	1189307			*WDR27*
**P53**	5m	F	Referred as obese	Dup	10p15.3 p12.31	119794-19509585	19389791	Not mat	Complex rearrangement	***ZMYND11*** *,* DIP2C *,* * IDI1 *,* ADRAB2 *,* PFKP *,* * KLF6 *,* ARK1C1 *,* AKR1C2 *,* ARK1C3 *,* AKR1C4 *,* UCN3 *,* IL15RA *,* IL2RA *,* * PFKFB3 *,* PRKCQ *,* ***GATA3,*** CDC123 *,* CAMK1D *,* CCDC3 *,* PTER *,* * CUBN *,* MRC1 *,* CACNB2
				Dup	13q11 q12.3	19440913-31031907	11590994	Not mat		***TUBA3C*** *,* ***GJB2*** *,* ***CRYL1*** *, SGCG,* ***SACS,*** *MIPEP,* GPR12 *,* GTF3A *,* MTIF3 *,* * POLR1D *,* PDX1 *,* * CDX2 *, POMP* , *SLC46A3* , *MTUS2* , *SLC7A1* , *UBL3,* KATNAL1, *LINC00426*
**P54**	9y	F	BMI 34.3 (99.7th)	Dup	10q26.11 q26.3	120306959-135434409	15127450	De novo	10qter duplication	PRLHR *,* * PRDX3 *,* ***BAG3*** *,* WDR11 *,* FGFR2 *,* * ACADSB *,* BUB3 *,* OAT *,* TCERG1L *,* PRAP1 *,* CYP2E1 *
**P55**	7y	F	BMI 23.6 (98.8th)	Del	12q15 q21.1	70555659-73153191	2597532	De novo	Clinically relevant	PTPRB *,* TSPAN8 *,* * LGR5TPH2
**P56**	13y	F	BMI 36.8 (99.4th)	Dup	12q21.32 q23.1	88684581-101464859	12780278	De novo	Insertional translocation	KITLG *,* ATP2B1 *,* SOCS2 *,* LTA4H *,* RMST *,* NR1H4 *
**P57**	15y	M	BMI 43.1 (99.7th)	Del	13q33.2 q34	106648660-115105655	8456995	Not mat	13qter deletion	***EFNB2*** *,* MYO16 *,* IRS2 *,* * COL4A1 *,* ***ARHGEF7*** *,* F7 *,* GAS6 *, CHAMP1*
**P58**	4y	M	BMI 22.0 (99.9th)	Dup	14q11.2	21244696-22250879	1006183	De novo	14q11.2 microduplication	***SUPT16H*** *,* ***CHD8***
**P59**	16y	F	BMI 38.5 (99th)	Del	14q12	29781404-30552936	771532	De novo	14q12 deletion, non-critical	PRKD1 *
				Dup	4p16.1	10068064-10529023	460959	Mat	Likely benign	***WDR1***
**P60**	7y	F	BMI 24.8 (99.3th)	Del	Xp22.12 p22.13	18214020-19833634	1619614	Unk	Rett syndrome-like	***CDKL5*** *,* ***RS1*** *,* PHKA2 *,* PDHA1 *,* * SH3KBP1
**P61**	14y	M	BMI 37.0 (99.5th)	Dup	Xp22.3	75943-2685605	2609662	De novo	Complex rearrangement	***SHOX*** , ***ASMTL*** , ***ASMT***
				Dup	Xq21.31 q21.32	88489522-92357353	3867831			*TGIF2 LX* , *PABPC5-AS1* , *PABPC5* , *PCDH11X*

*Abbreviations: M* male, *F* female, *Del* deletion, *Dup* duplication, *y* years, *m* months, *BMI* body mass index, *SD* standard deviation, *unk* unknown, *mat* maternally inherited, *pat* paternally inherited, *not mat* not maternally inherited; Genes in bold were listed in the CNV morbidity map of IDDs [34]. Underlined genes were retrieved from the Text-mined Hypertension, Obesity and Diabetes candidate gene database (T-HOD), the Human Genome Epidemiology (HUGE) Phenopedia, and from a list of obesity candidate genes curated from the literature [33]. Genes found at the intersection of at least two gene sets are highlighted (asterisks). Patients 6-12, 14, 15, 18, 23, 28, 30-33, 35, 40-43, 46-48, 51, 52, 55, 56, 58-60) have been published previously as separate studies [36-38]

#### Pathogenic CNVs

A total of 68 pathogenic imbalances were detected in 61 patients, the majority of which pathogenic deletions (72%) and rearrangements smaller than 5-Mb (70%). 31 of the patients (6–12, 14, 15, 18, 23, 28, 30–33, 35, 40–43, 46–48, 51, 52, 55, 56, 58–60) had previously been published as separate studies [36–38]. De novo PCNVs were found in 30 patients, whereas only 6 patients inherited a pathogenic deletion or duplication from an apparently unaffected parent, all of which occurring at genomic *loci* which are known *to* have reduced penetrance (16p13.11, 16p11.2, and 22q11.2)*.* The inheritance status could not be determined in 25 cases.

**Fig. 3:**
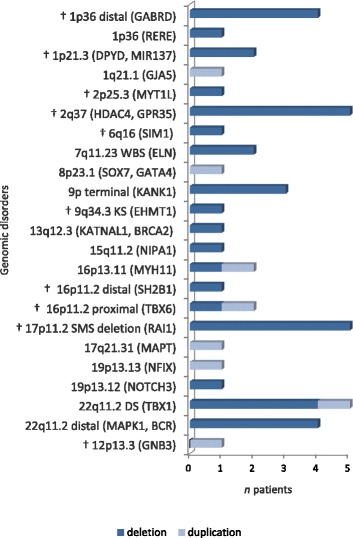
Known genomic imbalance disorders detected in patients. Imbalances that are known to be causal for syndromic obesity are indicated (stars). The genes in parentheses are identifiers of genomic locations. WBS: William-Beuren syndrome; SMS: Smith-Magenis syndrome; DG: DiGeorge syndrome; KS: Kleefstra syndrome

**Fig. 4:**
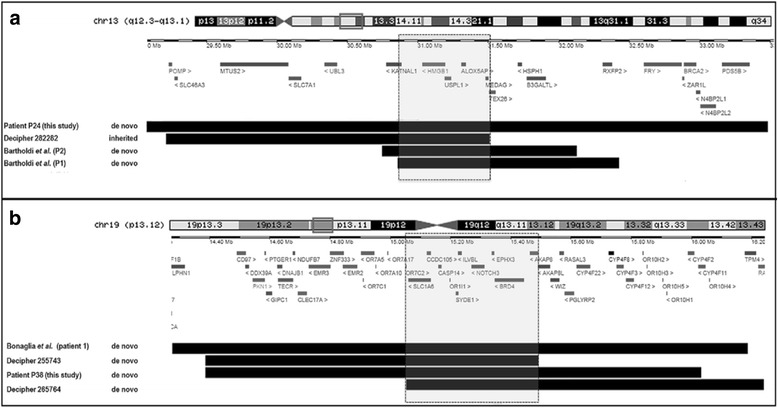
Schematic alignment using the UCSC Genome Browser custom track tools (hg19) showing the regions of overlap between the deleted segments detected in (A) patient 24 (this study), DECIPHER case 282,282, and patients 1 and 2 of Bartholdi’s study [69]; (B) patient 38 (this study), DECIPHER case 255,743 and 265,764, and patient 1 of Bonaglia’s study [71]. The inheritance of the CNVs and the protein-coding genes located within them are shown. The inner light gray boxes show the smallest regions of overlapping deletions

In 47 patients (24 novel cases), the PCNVs overlapped with chromosomal regions associated with known genomic disorders, and, among them, 35 patients were detected with PCNVs at 10 *loci* that were recurrent (same breakpoints) or overlapping in 2 or more unrelated samples (Fig. 3). We found 23 cases with deletions known to cause a syndromic obesity phenotype: 1p36 (*n* = 5), 1p21.3 (*n* = 2), 2p25.3 (*n* = 1), 2q37 (*n* = 5), 6q16 (*n* = 1), 9q34.3 (*n* = 1), 16p11.2 breakpoint (BP) 2–3 (*n* = 1), 16p11.2 BP 4–5 (*n* = 1), and 17p11.2 (*n* = 5). The recurrent translocation t(8;12)(p23.1;p13.31) found in patient 18 is also known to be involved in the pathogenesis of syndromic obesity. In addition, 2 rare deletions at chromosomes 13q12.3 (patient 24) and 19p13.12 (patient 37) overlapped with deletions of different sizes in patients from the literature and the DECIPHER database who were obese (Fig. 4). Mapping of the shortest region of overlap (SRO) in these cases exposed a 660-kb interval at 13q12.3 (chr13:30,880,255–31,540,272 bp, hg19; Fig. 4a) comprising 5 genes (*KATNAL1*, *LINC00426*, *HMGB1*, *USPL1*, *ALOX5AP*, and *MEDAG*), and a 440-kb interval at 19p13.12 (chr19:15,052,889–15,492,848 bp, hg19; Fig. 4b) comprising 9 genes (*SLC1A6*, *CCDC105*, *CASP14*, *SYDE1*, *ILVBL NOTCH3*, *EPHX3*, *BRD4*, and *AKAP8*).

**Fig. 5:**
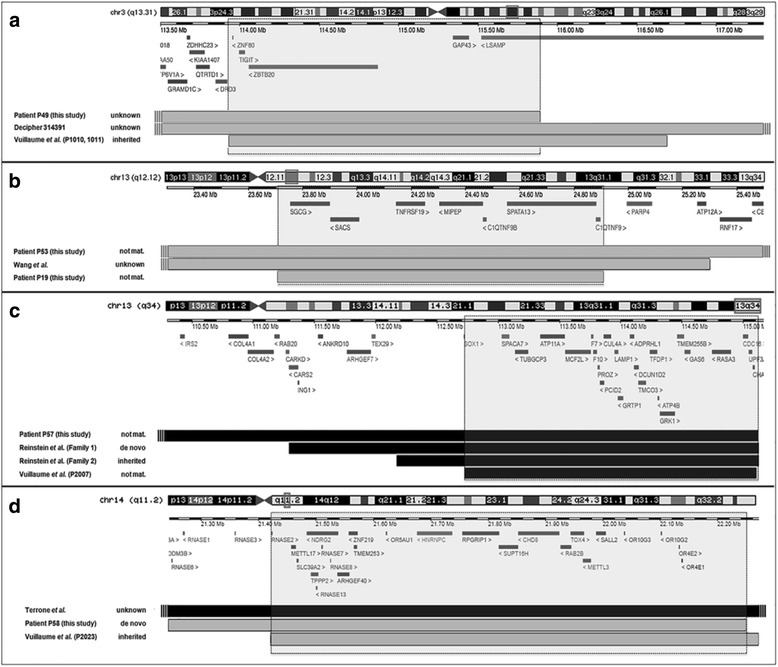
Schematic alignment using the UCSC Genome Browser custom track tools (hg19) showing the regions of overlap between deleted (black) and duplicated (grey) segments detected in (**a**) patient 19 (this study), DECIPHER case 314,391 and brothers P1010 and P1011 of Vuillaume’s study [22]; (**b**) patients 19 and 53 (this study) and one individual described by Wang et al. [75]; (**c**) patient 57 (this study), case P2007 of Vuillaume’s study [22] and families 1 and 2 of Reinstein’s study [21]; (**d**) patient 58 (this study), case P2023 of Vuillaume’s study [22], and one patient described by Terrone et al. [14]. Vertical lines depict breakpoints that extended beyond the regions indicated here. The inheritance of the CNVs and the protein-coding genes located within them are shown. The inner light gray boxes show the smallest regions of overlap

In other 14 patients, the PCNVs did not overlap with a known genomic imbalance disorder but were de novo in 10 cases or of unknown origin (the remainder), and involved large and complex chromosomal imbalances. Among these, we found 4 PCNVs overlapping with previously identified obesity candidate *loci* (Fig. 5): a novel 22.3-Mb duplication of 3q11.2q13.31 (patient 49), a novel 11.6-Mb duplication of 13q11q12.3 (patient 53), a novel 8.5-Mb deletion of 13q33.2q34 (patient 57), and a 1-Mb duplication of band 14q11.2 (patient 58). Notably, the duplication region at 13q11q12.3 also overlaps with a smaller duplication present in our patient 19, who possessed a second large CNV at the 8p23.1 *locus*. The extent of overlap among our cases with those previously described CNVs is of about 2-Mb in band 3q13.31 (chr3:113,924,534–115,890,384 bp, hg19; Fig. 5a), 1.2-Mb in band 13q12.12 (chr13:23,706,634–24,910,765 bp, hg19; Fig. 5b), 2.4-Mb in band 13q34 (chr13:112,725,394–115,092,648 bp, hg19; Fig. 5c), and 827-kb in band 14q11.2 (chr14:21,424,185–22,250,879 bp, hg19; Fig. 5d). Candidate genes at these intervals are proposed in the discussion.

#### Likely pathogenic CNVs

CNVs detected in 6 additional patients were classified as potentially clinically significant, including a de novo 340-kb duplication at 16p13.2 comprising the *USP7* gene implicated in a known deletion syndrome (OMIM #616863), a 482-kb paternally inherited duplication of 17q11.2 partially overlapping the gene for neurofibromatosis type I (NF1), and an 1.1-Mb paternally inherited 20q11.2 duplication upstream of the *ASXL1* gene with a likely role in 20q11.2 duplication syndrome [39]. The inheritance of 3 other CNVs could not be determined. The 3 CNVs were a 489-kb duplication in 21q22.13 including the ID gene *DYRK1A* (OMIM #614104), a 703-kb deletion in 7q31.1 affecting the *IMMP2L*-*DOCK4* gene region implicated in IDDs [40], and an intragenic deletion of the *ASTN2* gene at 9q33.1 which has also been implicated in susceptibility to IDDs [41].

#### Variants of uncertain significance

In 15 patients, we detected CNVs that were classified as VUS. Of these, 2 were de novo events: a 222-kb 12q21.32 duplication including the *CEP290* gene whose mutations cause Bardet-Biedl syndrome (BBS14; OMIM #615991), but which was not associated with previously reported pathogenicity, and a 385-kb 6q27 duplication affecting only three non-coding RNAs found in a patient who inherited a second large CNV. There were 3 VUS inherited from asymptomatic parents intersecting with genes within CNVs that have previously been implicated with disorders, such as *CACNA2D1* with epilepsy and ID [42], and *MACROD2* and *LINGO2* with autism [43, 44]. Furthermore, 6 VUS (3 inherited and 3 unknown) contained morbid OMIM genes, including *NFIA*, *MPZ*, *PARK2*, *DPP6*, and *KANK1*. Additionally, 3 other cases (2 females and 1 male) inherited large chromosome X duplications from carrier mothers spanning several morbid OMIM genes but with no evidence for triplosensitivity phenotypes as determined by the ClinGen Dosage Sensitive Map (http://www.ncbi.nlm.nih.gov/projects/dbvar/clingen/) [45].

#### Likely benign CNVs

We also observed 30 patients with CNVs that might represent benign variants. The observed CNVs were most often duplications and < 300-kb in size. In all cases where it was possible to ascertain the parental status, variants were inherited from an asymptomatic parent. We found relevant genes lying within some of these CNVs. Examples of such genes include: *PTEN* in which mutations cause many different disorders including macrocephaly/autism syndrome (OMIM #605309); *VPS13B* whose mutations cause Cohen syndrome characterized by truncal obesity, joint hypermobility and a pigmentary retinopathy (COH1; OMIM #216550); *CIDEA* (OMIM 604440) with a role in regulating energy balance and adiposity; *ULK4* crucial to brain development with CNVs being identified as risk factor in schizophrenia [46]; *KATNAL2* implicated as susceptibility gene of autism [47].

### Identification of candidate genes involved in obesity susceptibility

**Fig. 6:**
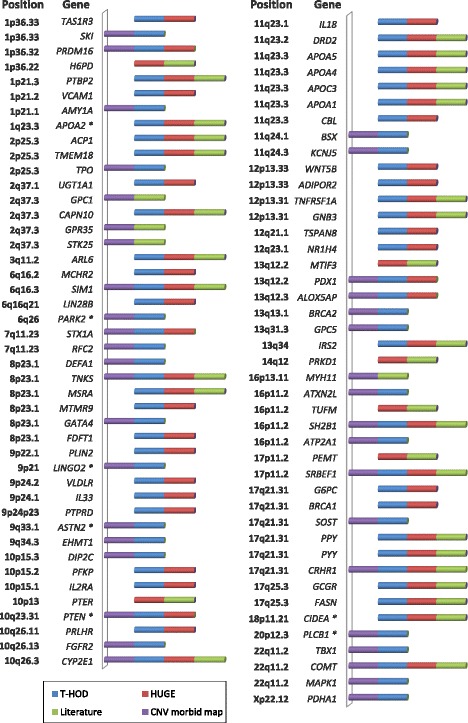
shows 87 prioritized genes and their positions within PCNVs, but also likely PCNVs, VUS and benign CNVs (asterisk). Prioritized genes were retrieved by at least 2 independent gene sets, including obesity candidate genes collected from the T-HOD (Text-mined Hypertension, Obesity and Diabetes) and HUGE (Human Genome Epidemiology) database, a list of genes with evidence for playing a role in obesity curated from literature [33], and genes in the CNV morbidity map for IDDs [34]

A total of 2684 genes were affected by the detected CNVs. Among these, 234 genes had some previously reported connection to obesity as determined by the overlap with genes from the T-HOD and HUGE database, as well as a gene list curated from literature [33], and 172 overlapped with genes listed in the CNV morbidity map for IDDs [34] (367 genes in total). Of particular interest are 87 genes that were intersected by different gene sets (Fig. 6). Notably, several known and candidate genes that have previously been implicated in syndromic obesity were retrieved by this candidate gene approach, including *SIM1* [48], *SH2B1* [49]*, PTBP2* [38]*, PRLH* and *CAPN10* [50], *ACP1* and *TMEM18* [12]*, EHMT1* [51]*,* and *GNB3* [17]. Thus, these genes were considered as more likely to have a role in obesity susceptibility. Pathways analysis in Cytoscape using the plugin Reactome FI showed that the majority of these genes were related to metabolic pathways and small molecule metabolic process.

### PCNV rates by gender, age and level of obesity in children and adolescents

**Fig. 7:**
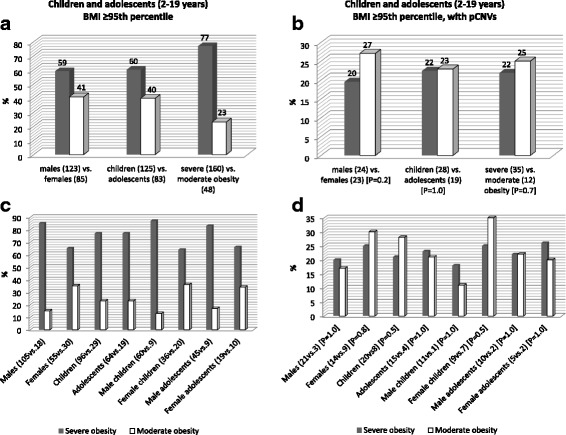
(A) Frequencies of children and adolescents in the whole cohort with BMI ≥ 95th percentile stratified by gender (males and females), age groups (2–9 years and 10–19 years), and level of obesity (moderate and severe). (B) Sex- and age-related differences of children and adolescents in the whole cohort by level of obesity. (C, D) same comparisons in children and adolescents with PCNVs. The numbers in parentheses indicate the total number of patients in each group. Definition of moderate and severe obesity was based on the BMI below or above 120% of the 95th percentile. *P*-values for the Fisher’s exact test are shown

For the purpose of this study, 208 obese children and adolescents with a BMI ≥95th percentile were considered based on a percentage above the 95th BMI percentile as moderately (< 120% of the 95th percentile) and severely (≥120% of the 95th percentile) obese (Fig. 7a). This sample consisted predominantly of males (*n* = 123 or 59%) and children aged 2–9 years (*n* = 125 or 60%). The majority of patients were classified as severely obese (*n* = 160 or 77%). The prevalence of severe obesity was higher than moderate obesity for both gender and age groups (Fig. 7b) but there were statistically significantly more males than females with severe obesity (105 males, 55 females; Fisher’s Exact test, *p* < 0.01); this sex-related difference in obesity was observed in children but not in adolescents aged 10–19 years (Additional file 4: Table S4). Although a higher frequency of PCNVs was observed in females (*n* = 23 or 27%) compared with males (*n* = 24 or 20%), no statistically significantly differences were observed in the frequencies when compared by gender, age at the time of testing, and obesity severity (Fig. 7c and d; Additional file 5: Table S5). In boys, the prevalence of PCNVs was greater in the severe obesity group (20% vs. 17%), particularly among children aged 2–9 years (18% vs. 11%), but was similar at adolescence (22%). In girls, the prevalence of PCNVs was greater among the moderate obesity group (35% vs. 30%), particularly among children aged 2–9 years (35% vs. 25%), and correlated inversely in adolescents (26% severe vs. 20% moderate obesity).

## Discussion

This is the second study describing the use of CMA in patients ascertained for syndromic obesity, the largest published to date and also the first in a Brazilian cohort. We identified PCNVs in 22% of patients (68 pathogenic events in 61/279 subjects; Table 3), which is similar to the yield reported by Vuillaume et al. in microarray studies with 100 patients with syndromic obesity [22]. The prevalence of PCNVs in children and adolescents did not differ significantly between gender and age groups, and obesity severity (Fig. 7). Nevertheless, females had a higher detection rate of PCNVs in comparison to males (27% females and 20% males; overall), with the highest differences (35% females and 11% males) found in the younger age group (2–9 years) with less severe grades of obesity (BMI < 1.2 x 95th percentile). Whilst no single phenotypic feature could be investigated for association with PCNV risk, due to the absence of comprehensively phenotyping of patients, phenotype-phenotype correlation analysis between cases with and without PCNVs identified 12 pairs of phenotypes that were significantly associated with the presence of PCNVs and combining hypotonia, language impairments, abnormalities of the external genitalia, and eye/vision problems at its core. Of note, patients in our cohort were almost 10 times more likely to manifest macrocephaly as compared to microcephaly. Even though 33% of macrocephalic patients displayed PCNVs, macrocephaly did not associate with the presence of PCNVs.

In the current study, we have identified known genomic imbalance disorders in 47 patients, and, of them, 35 patients (13%) carry overlapping and recurrent CNVs (Table 3; Fig. 3). In our cohort, imbalances that are known to be causal for syndromic obesity were observed in 23 patients (8.2%). The most commonly identified syndromic forms of obesity were deletions of the chromosomal regions 1p36, 2q37 and 17p11.2, which collectively represented 5.4% of all cases, followed by microdeletions of the 1p21.3 region (2 cases). In 6 other syndromic obesity *loci* (2p25.3, 6q16, 9q34, 16p11.2 proximal and distal, 12p13.31), CNVs were found only in one unrelated individual. The identification of CNVs overlapping *loci* previously shown to be involved in syndromic obesity further implicates them as risk factors for obesity. As previously mentioned, *SH2B1*, *SIM1*, *PTBP2, PRLH*, *CAPN10, ACP1, TMEM18, EHMT1,* and *GNB3* are relevant candidate and known genes for obesity within these regions (Fig. 6), and *POU3F2* [20], *HDAC4* [50], *MYT1L* [52], and *RAI1* [53] were also candidate genes identified in these *loci*. Moreover, our gene prioritization analysis identified 20 new genes of interest to obesity overlapping these CNVs, among which we highlight the potential importance of *TAS1R3*, encoding a taste receptor differentially expressed in obese mice [54]. This gene maps within the common deleted region of patients with distal 1p36 deletion.

In addition to the above, we identified 4 patients with recurrent deletions at the 22q11.2 DiGeorge syndrome (DS) region (we also found a patient with duplication of the same region), 4 patients with distal 22q11.2 recurrent deletions, and 3 patients with overlapping deletions at 9p terminal. As these CNVs arise in more than 2 unrelated individuals, we implicate them as novel *loci* with a potential role in obesity susceptibility. A link between the 22q11.2 region with obesity is also supported by previous works showing that 22q11.2DS deletion carriers have increased rates of obesity [55–57], as well as reports of patients presenting childhood obesity with hyperphagia [58, 59]. Overweight and obesity (with or without hyperphagia) have also been described in a number of patients with distal 22q11.2 deletions [60–63]. We identified 3 genes at 22q11.2 (*TBX1*, *COMT* and *MAPK1*) that could confer susceptibility to obesity (Fig. 6). Although obesity is not a reported feature of deletion 9p syndrome, weight ≥ 90th percentile at birth or in childhood was documented in 4 of a series of 10 patients with distal deletions of 9p [64], further emphasizing the potential importance of this region. Additionally, we recently detected a deletion at 9p24.3p24.2 in one further patient with syndromic obesity using multiplex ligation-probe amplification (unpublished data from our laboratory). The *VLDLR*, *IL33* and *PTPRD* genes were identified as the most interesting genes for obesity-susceptibility within 9p24 (Fig. 6). Furthermore, we detected 2 patients with Williams-Beuren syndrome (WBS) 7q11.23 deletions. This region was already shown to be associated with several endocrine and metabolic problems including hypothyroidism, hypercalcemia, obesity and diabetes [65, 66]. Two genes related to obesity, *STX1A* and *RFC2*, map to this CNV interval (Fig. 6).

In this study we discovered recurrent CNVs at 1q21.1 and 16p13.1, which are known predisposing factors to IDDs reported sometimes in patients exhibiting obesity [67, 68]. Moreover, CNVs at these *loci* were also documented in a cohort study of syndromic obesity [22]. We also discovered other CNVs overlapping *loci* involved in syndromic obesity cases from the literature and DECIPHER. For instance, patient 24 carry a deletion overlapping the critical region of the 13q12.3 microdeletion syndrome described by Bartholdi et al. in 3 unrelated patients, two of whom with obesity [69]. This deletion was further associated with obesity in a patient from DECHIPER (case 282,282). Five genes map to the common CNV interval (Fig. 4a), among them *ALOX5AP* whose expression was linked to obesity and insulin resistance [70]. Likewise, patient 38 carry a deletion at 19p13.12 partially overlapping with those reported in 3 patients from literature, one of them with obesity [71]. We found 2 other patients in DECIPHER with deletions at this *locus* in addition to obesity (cases 255,743 and 265,764). These cases share a 440-kb SRO encompassing 9 genes, including *NOTCH3* (Fig. 4b). The Notch signaling has recently emerged as a key player in regulating metabolism [72]. We also identified a case of 19p13.2 duplication involving the *NFIX* gene associated with a Sotos syndrome-like phenotype [73]. This CNV was associated with a 430-kb 9p22.1 duplication that encompassed the entire *PLIN2* gene, which is involved in the control of energy balance [74]. Notably, CNV in this gene has previously been identified in a patient with syndromic obesity [22].

Other than CNVs overlapping known genomic disorders *loci*, 14 patients had other chromosomal defects that are known to be clinically relevant, among which 4 overlapped with *loci* previously implicated in obesity. The distal portion of the 3q11.2q13.31 duplication in patient 49 (Fig. 5a) partially overlaps with a 2.76-Mb 3q13.31 duplication found in 2 brothers with syndromic obesity [22], and with a 9.8-Mb 3q13.13q13.32 duplication reported in association with obesity from DECIPHER (case 314,391). The common region of overlap involves 5 genes and among them *ZBTB20* is implicated in Primrose syndrome associated with several endocrine features and obesity (OMIM #259050). The large 13q11q12.3 duplication in our patient 53 (Fig. 5b) overlaps with a 1.2-Mb 13q12.12 duplication also found in our patient 19 and with another 2-Mb 13q12.11q12.12 duplication detected among cases with moderate and extreme obesity [75]. This region involves the gene *SGCG* with expression in adipose tissues and associated with type 2 diabetes [76]. Patient 57 carry an 8.5-Mb 13q33.2q34 deletion encompassing the ID gene *CHAMP1* (Fig. 5c), which partially overlaps with deletions at 13q34 found in 6 other patients with syndromic obesity reported by Vuillaume et al. [22] and Reinstein et al. [21]. Of interest, the deleted region in each of the 7 cases overlaps the obesity-associated gene *GAS6* [77]. The 14q11.2 microduplication found in our patient 58 (Fig. 5d), including the *SUPT16H* and *CHD8* genes, was also identified in one patient with syndromic obesity reported by Vuillaume et al. [16]. No candidate genes for obesity were associated with this *locus*. Although there is one case of 14q11.2 deletion that was reported with severe obesity, it included a large more proximal segment of 14q11.2, which contains a strong obesity candidate gene [14].

Finally, in a total of 51 patients the CNVs were classified as potentially pathogenic (2.1%), VUS (4.7%) or likely benign variants (11.5%). Overall, a number of interesting genes that could play a role in obesity susceptibility have been identified within these CNVs (e.g. *ASTN2, APOA2*, *PARK2*, *LINGO2*, *PLCB1*, *PTEN*, and *CIDEA*). More importantly, we identified a new and de novo 340-kb 16p13.2 duplication that encompasses the entire *USP7* gene. Although its pathogenicity is not certain, since similar duplications have not been reported in the literature, there is one reported patient with larger duplication at the *USP7* locus presenting with severe early-onset obesity and hyperphagia [78]. Of note, USP7 has been identified as an integral component of MAGEL2 and TRIM27 ubiquitin ligase complex, which plays an important role in hypothalamic function [79]. Moreover, deletion or mutation of *USP7* has been shown to result in a neurodevelopmental disorder with overlapping symptoms to Schaaf-Yang syndrome (OMIM #615547), caused by mutations of *MAGEL2* [78]. There are 5 de novo duplication events overlapping *USP7* (400-kb to 1.2-Mb) reported in DECIPHER with no additional changes detected. These included 3 patients (cases 269,501, 281,449 and 258,037) with delayed speech and language development as common features and 2 patients (cases 254,000 and 267,094) with no phenotypic description. One of the limitations of our study is that additional independent risk factors were not considered, including unidentified genetic factors and those being epigenetic, environmental, or stochastic in origin. Future investigations of genes within disease-specific CNVs detected in the present cohort are also needed. Future directions will involve whole exome sequencing (WES) in patients that did not reach a diagnosis to estimate the contribution of single gene mutations in the genetic causation of syndromic obesity. This will allow isolate genes that cause or may affect susceptibility to obesity in humans, advancing our understanding of the molecular mechanisms involved in body weight regulation and provide clues for therapeutic intervention in obesity.

## Conclusion

Understanding the genetics of obesity has proven difficult. Although it is likely that not all of the PCNVs detected in the current study are directly causative of obesity, we found that 23/279 (8.2%) of our patients carried rare CNVs at 10 *loci* already known to increase the risk of obesity. We identified 3 patients with overlapping deletions at 9p terminal, 4 patients with deletions of 22q11.2DS and 4 patients with deletions at distal 22q11.2, which thus emerge as new putative obesity-susceptibility *loci*. In addition, we found that CNVs in at least 6 other cases overlapped with *loci* previously implicated in syndromic obesity, including a new patient with deletion at chromosome 13q34. This *locus* is particularly interesting because our new case brings to 7 the number of patients in whom such defects have been described in association with obesity. Overall, we found CNVs that further implicate genes previously associated with obesity such as *PTBP2*, *TMEM18*, *MYT1L*, *POU3F2*, *SIM1*, *SH2B1* and *GNB3*, and also identified other potentially relevant candidate genes including *TAS1R3*, *ALOX5AP*, and *GAS6.* Our study highlights the significant value of chromosomal microarrays in providing not only a genetic diagnosis for syndromic causes of obesity but in uncovering genes relevant to human obesity.

## Additional files

Additional file 1: Table S1. Full description of clinical findings in patients enrolled in this study. (XLSX 71 kb)

Additional file 2: Table S2. Frequencies of phenotypes pairs segregating together in patients with and without pCNVs. (XLSX 18 kb)

Additional file 3: Table S3. Likely PCNVs, VUS and likely benign variants detected in patients with syndromic obesity (XLSX 19 kb)

Additional file 4: Table S4. Evaluation of the level of obesity in 208 children and adolescents with BMI ≥ 95th percentile. (PDF 81 kb)

Additional file 5: Table S5. Comparison of the pCNVs rates for children and adolescents with BMI at or above the 95th percentile by age, sex and level of obesity. (PDF 81 kb)

## Abbreviations

ACMG: American college of medical genetics
ASD: Autism spectrum disorder
BBS: Bardet-Biedl Syndrome
BMI: Body mass index
BP: Breakpoint
CDC: Centers for disease control and prevention
ClinGen: Clinical genome resource
CMA: Chromosomal microarray analysis
CNV: Copy number variant
COH1: Cohen syndrome
DECIPHER: DatabasE of genomiC varIation and phenotype in humans using ensembl resources
DG: DiGeorge syndrome
DGV: Database of genomic variants
HUGE: Human genome epidemiology
ID: Intellectual disability
IDD: Intellectual and developmental disabilities
ISCA: International standards for cytogenomic arrays
KS: Kleefstra syndrome
OFC: Occipitofrontal circumference
OMIM: Online mendelian inheritance in man
PCNV: Pathogenic copy number variant
PWS: Prader-Willi syndrome
SD: Standard deviation
SMS: Smith-Magenis syndrome
SRO: Smallest region of overlap
T-HOD: Text-mined hypertension, obesity and diabetes
VUS: Variants of uncertain significance
WBS: Williams-Beuren syndrome
WES: Whole-Exome sequencing
