# Supplementary material for: Chromosomal microarray analysis in the genetic evaluation of 279 patients with syndromic obesity
Source: Mol Cytogenet. 2018 Feb 5;11:14. doi: 10.1186/s13039-018-0363-7 (PMC5800070; doi:10.1186/s13039-018-0363-7)
Supplement: Supplementary file 4 — Evaluation of the level of obesity in 208 children and adolescents with BMI ≥ 95th percentile. (PDF 81 kb) [file 13039_2018_363_MOESM4_ESM.pdf]

Table S4: Evaluation of the level of obesity in 208 children and adolescents with BMI ≥ 95th percentile

Groups	Severe obesity	Moderate obesity	Fisher's Exact test
Males (%)	105/123 (85)	18/123 (15)	**p=0.0007**
Females (%)	55/85 (65)	30/85 (35)	
Children (%)	96/125 (77)	29/125 (23)	p=1.0
Adolescents (%)	64/83 (77)	19/83 (23)	
Male children (%)	60/69 (87)	9/69 (13)	**p=0.005**
Female children (%)	36/56 (64)	20/56 (36)	
Male adolescents (%)	45/54 (83)	9/54 (17)	p=0,1
Female adolescents (%)	19/29 (66)	10/29 (34)	

Children (2-9 years)

Adolescents (10-19 years)

Severe obesity (≥120% of the 95th)

Moderate obesity (<120% of the 95th)

The fractions in parentheses indicate the number of cases that manifested the phenotype over the total number of cases.
