# Supplementary material for: Chromosomal microarray analysis in the genetic evaluation of 279 patients with syndromic obesity
Source: Mol Cytogenet. 2018 Feb 5;11:14. doi: 10.1186/s13039-018-0363-7 (PMC5800070; doi:10.1186/s13039-018-0363-7)
Supplement: Supplementary file 5 — Comparison of the pCNVs rates for children and adolescents with BMI at or above the 95th percentile by age, sex and level of obesity. (PDF 81 kb) [file 13039_2018_363_MOESM5_ESM.pdf]

**Table S5**: Comparison of the pCNVs rates for children and adolescents with BMI ≥95th percentile by age, sex and level of obesity

| Groups | Severe obesity | Moderate obesity | Fisher's Exact test |
|---|---|---|---|
| Males (%) | 21/105 (20) | 3/18 (17) | p=1.0 |
| Females (%) | 14/55 (25) | 9/30 (30) | p=0.8 |
| Children (%) | 20/96 (21) | 8/29 (28) | p=0.5 |
| Adolescents (%) | 15/64 (23) | 4/19 (21) | p=1.0 |
| Male children (%) | 11/60 (18) | 1/9 (11) | p=1.0 |
| Female children (%) | 9/36 (25) | 7/20 (35) | p=0.5 |
| Male adolescents (%) | 10/45 (22) | 2/9 (22) | p=1.0 |
| Female adolescents (%) | 5/19 (26) | 2/10 (20) | p=1.0 |

Children (2-9 years)

Adolescents (10-19 years)

Severe obesity (≥120% of the 95th)

Moderate obesity (<120% of the 95th)

The fractions in parentheses indicate the number of patients with pCNVs over the total number of cases in each group.
